# A Delicate Nanoscale Motor Made by Nature—The Bacterial Flagellar Motor

**DOI:** 10.1002/advs.201500129

**Published:** 2015-06-25

**Authors:** Ruidong Xue, Qi Ma, Matthew A. B. Baker, Fan Bai

**Affiliations:** ^1^ Biodynamic Optical Imaging Center (BIOPIC)School of Life SciencesPeking UniversityBeijingP. R. China; ^2^ Victor Chang Cardiac Research InstituteSydneyNSWAustralia

**Keywords:** molecular motor, bacterial flagellum, bacterial motility, chemotaxis, nanobiotechnology

## Abstract

The bacterial flagellar motor (BFM) is a molecular complex ca. 45 nm in diameter that rotates the propeller that makes nearly all bacteria swim. The motor self‐assembles out of ca. 20 different proteins and can not only rotate at up to 50 000 rpm, but can also switch rotational direction in milliseconds and navigate its environment to maneuver, on average, towards regions of greater benefit. The BFM is a pinnacle of evolution that informs and inspires the design of novel nanotechnology in the new era of synthetic biology.

## 1 Introduction

In an aqueous environment, bacteria benefit from swimming to where life is better, and most bacteria swim by rotating their flagella. This rotation is powered by the electrochemical ion motive force arising from the transit of ions across the cellular membrane.^1^, ^2^ This provides energy to free‐swimming bacteria to propel their cell body at a speed of 15–100 μm s^−1^, or up to 100 cell body lengths per second. The BFM is a nanoscale rotary molecular machine embedded in the cell envelope and possesses an outstandingly efficient mechanochemical conversion between electrochemical free energy and mechanical work. A single BFM in *Escherichia coli* can output a power of ca. 1.5 × 10^5^ pN nm s^−1^ 3 and rotate at ca. 300 Hz (18 000 rpm)^4^ while the BFM in *Vibrio alginolyticus* can rotate as fast as ca. 700 Hz (42 000 rpm),^5^ which is nearly triple the 15 000 rpm of a modern Formula 1 racer. Additionally, while man‐made machines suffer from energy loss due to heating, the BFM operates at near 100% efficiency of energy conversion from ion transit to motor torque.^6^

Motility at low Reynolds numbers requires changes in the manner of propulsion when compared with inertial regimes. First, propulsion requires a time‐irreversible swimming motion, which is provided by the helical nature of the propeller (the filament), and, secondly, a bacterium must be able to outrun diffusion in order to be able to swim, on average, towards where life is better. The BFM can rotate in two directions, counterclockwise (CCW) and clockwise (CW). When all motors on an *E. coli* cell spin CCW, all of its flagellar filaments form a bundle to push the cell steadily forward (run state); when one or more of the motors switch to spin CW, the flagellar bundle breaks apart and the cell tumbles randomly due to diffusion (tumble state). The BFM switches stochastically between CCW and CW and the cell repeats a “run”–“tumble”–“run” pattern, which enables chemotactic navigation of its environment (reviewed in ref. 7. Bacterial response to chemical stimuli is known as chemotaxis,^8^ first demonstrated by T.W. Engelmann in 1881 (reviewed in ref. 1 pages 7–11). The BFM not only drives bacterial locomotion, it also plays a crucial role in bacterial chemotaxis by controlling the ratio between CCW and CW rotation, and thus duration of run and tumble events.

A comprehensive study of the BFM not only helps us understand bacteria better, it enables the development of novel anti‐bacterials not derived from antibiotics.^9^ Understanding how this canonical complex self‐assembles informs the development of bespoke bionanotechnology by revealing the natural design principles of self‐organization. Here we use the BFM in *E. coli*, the best characterized BFM among all, as an example to introduce the intricacy of its structure, assembly, energetics, power generation, and switching mechanism.

## 2 Structure and Assembly of The Bacterial Flagellum

*E. coli* is a Gram‐negative, facultatively anaerobic bacterium, which is normally rod‐shaped, about 2 μm long and 1 μm in diameter. *E. coli* is able to grow and reproduce very quickly, with a doubling time of ca. 20 minutes.^10^ It can adapt and survive in variable growth conditions and has a small genome of 4.6 Mb that can be easily genetically manipulated.^11^, ^12^ For those reasons, *E. coli* has been chosen as the “model organism” for studying many essential cellular processes in prokaryotes.

**Figure 1:**
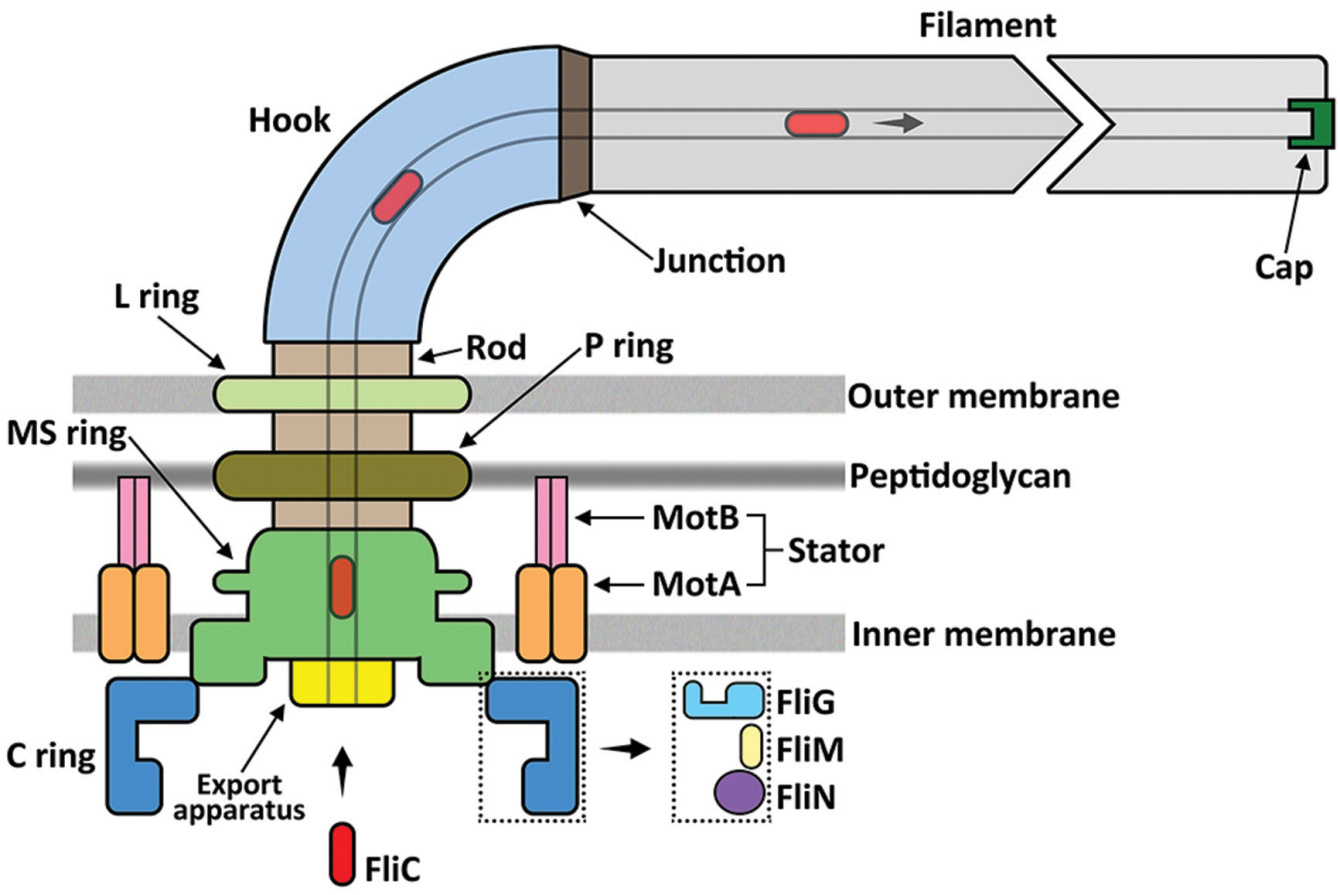
A schematic plot of the major structural components of the bacterial flagellum.

Each *E. coli* cell has about 4–5 flagella randomly distributed on the cell surface.^7^ A bacterial flagellum is composed of three parts: the BFM, the flagellar filament, and the hook connecting them (**Figure** **1**). A swimming bacterium is propelled by the fast rotation of these helical filaments, each driven at its base by a BFM through the hook.

The filament is the longest part of the bacterial flagellum and can extrude up to 10 μm from the cell body. It is a long helical hollow tube aligned by 11 parallel protofilaments, which are the polymers of FliC, the flagellin subunit.^13^ These flagellin molecules can bind in two different ways, resulting in protofilaments with different lengths.^14^ The flagellar filaments appear in different helical shapes because of various length combinations of the protofilaments. The conformational change of filament shape plays a key role in the switching between “tumble” and “run” states.^15^

The filaments are connected to the motor by a flexible module, named the hook. Mechanically, the flagellar hook works analogously to a universal joint. The hook is a short curved tube, assembled by 120 copies of hook protein unit—FlgE.^16^ The curvature of the hook remains constant when the bacterial flagellum rotates. Thus, the hook can transmit torque generated by the motor efficiently while rotating the flagellar filaments through an arc.^17^

**Figure 2:**
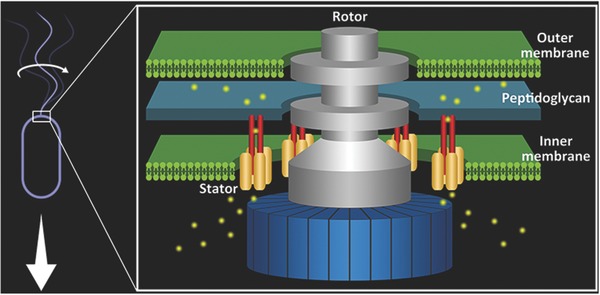
Rapid rotation of the flagellum is driven by the bacterial flagellar motor embedded in the cell envelope, harvesting the free energy of ion flux across the cytoplasmic membrane.

The central mechanical component, the bacterial flagellar motor, ca. 45 nm in diameter, with a total molecular mass of 11 MDa, is built by about 20 proteins and spans across the outer membrane, peptidoglycan wall and the inner membrane, into the cytoplasm.^18^ Similar to all rotary motors, its structural constituents can be divided into two categories: the rotor and the stators (**Figure** **2**).

On the rotor of the BFM, four concentric protein rings are conjugated by a rod in their center: the L‐ring, P‐ring, MS‐ring, and C‐ring, altogether comprising the basal body.^19^ These rings are named according to their locations within the membrane. The L‐ring sits at the lipopolysaccharide outer membrane, while the P‐ring is located at the peptidoglycan cell wall, and both are believed to have a bushing role between the motor and outer cell envelope. The MS‐ring stands for membrane and supramembrane ring and it is constructed by ca. 26 FliF protein subunits. The C‐ring is in the cytoplasm, mounted on the MS ring. It is composed of ca. 26 copies of FliG, ca. 34 copies of the FliM, and more than 100 copies of FliN proteins. The rod connects all the protein rings and is made by FlgB, FlgC, FlgF and FlgG. Altogether, this forms the rotor of the motor.

The stators of the BFM are located around the periphery of the rotor, forming a larger concentric protein ring outside the MS‐ring. The BFM can accommodate 8–12 stator complexes, each of which is made up of 4 MotA and 2 MotB proteins.^20^ MotA has a cytoplasmic domain interacting with the rotor to generate torque. MotB has a cytoplasmic domain and a linker domain that anchors the stator complex to the peptidoglycan cell wall. Two ion channels are formed on each stator complex that allow the passage of protons from the periplasm to the cytoplasm of the cell.^21^ Previous structural and biochemical studies indicate that torque is generated between the C‐terminal domain of the rotor protein FliG and the cytoplasmic domain of the stator protein MotA.^22^, ^23^ Both steric and electrostatic interactions are believed to be crucial for the torque generation, with proton flux coordinating conformational change in MotA, to drive rotation by direct interaction with FliG. Mutational studies revealed that a conserved Asp residue on the MotB, Asp 32, is essential for motor function, possibly providing the proton binding site in proton transfer.^24^ Critical charged residues have also been found on MotA and FliG, which play important roles in torque generation and motor function.^22^, ^25^

The assembly of the bacterial flagellum begins on the cytoplasmic side of the BFM. It is built from inside out and all the external proteins of the hook and the long filament proteins have to be exported by the flagellar type III secretion system, a close analogue of the type III virulence secretion system.^26^ When the BFM assembly commences, first the MS‐ring and the export apparatus are formed. Subsequently, the stators, rod, and other rings self‐assemble at their respective positions, after which the export apparatus located beneath the C‐ring delivers FlgE monomers through the hollow interior of the rod to assemble the hook at the cell's exterior. Once the hook is finished, the export apparatus switches to export FliC monomers to build the long filament.^27^ In this dynamic assembling process, FliD proteins serve as a cap at of the far end of the filament, coordinating the FliC polymerization and elongation of the filament.^28^ A key challenge here is to understand how protein substrates of different sizes can be exported by the same apparatus through a narrow channel (about 2 nm in diameter) in the flagellum.^14^ Previous studies have shown that these protein monomers are unfolded into peptide chains when entering the export gate;^29^ however, many details of this process remain poorly understood.^30^

## 3 Function of the Bacterial Flagellar Motor

The BFM coordinates precisely the organized motion of multiple proteins to propel bacteria. As such, the BFM shares common features with all motors, for instance the use of repeating cycles. However, due to distinctions in their working environments, the BFM necessarily functions differently to macroscopic engines.

The BFM is a nanoscale molecular motor whose environment is dominated by thermal fluctuations.^31^ A distinct feature of this environment is described as “the world of low Reynolds number”.^32^ At low Reynolds number, when small things move slowly through fluids, viscous forces are significant but inertial forces are not. Thus, a small object stops moving immediately if the driving force/torque is withdrawn. On the other hand, while Brownian motion blurs trajectories, it also provides a stochastic force that can cause molecules to pass over high energy barriers, unlike the scenario seen in the macroscopic world.

### 3.1 Power Input

Distinct from most linear molecular motors, such as myosin on actin filaments, kinesin and dynein on microtubules, which are powered by ATP hydrolysis, the BFM is powered by the free energy released from flow of ions down an electrochemical gradient across the cytoplasmic membrane into the cell (Figure 2). This energy source is termed the protonmotive force (PMF), which is maintained by the electron transport chain and ATPase involved in metabolic processes. Other bacteria, especially those living in marine or high pH environments consume Na^+^ rather than H^+^.

The PMF consists of two parts (Equation (1)): The first contribution is from the transmembrane electrical potential gradient, or the membrane potential. This arises from the electric field generated by different concentrations of cations and anions across the membrane. The second component consists of the entropic force arising from transmembrane concentration differences:

(1) $$\mathrm{PMF}=\Delta p=\Delta \psi +2.3\frac{k_{B}T}{e}\Delta pH$$

Here, Δψ represents the electrical potential across cytoplasmic membrane, Δ*pH* denotes the change in proton concentration across the membrane, *k_B_* is the Boltzmann constant, *T* the absolute temperature, and *e* the proton charge.

At room temperature and *E. coli'*s normal growth conditions, the internal pH of a bacterial cell is about 7.6–7.8. For *E. coli* grown at pH 7 and 24 °C, Δψ ≈ –120 mV, $2.3\frac{k_{B}T}{e}\Delta pH\approx$ –50 mV, Δp ≈ –170 mV.^7^

### 3.2 Power Output

Like macroscopic machines, the torque‐speed relationship is widely used to assess the performance of a molecular motor since it provides a full picture of the power output of the motor under different external loads, and also indicates the energy conversion efficiency.

**Figure 3:**
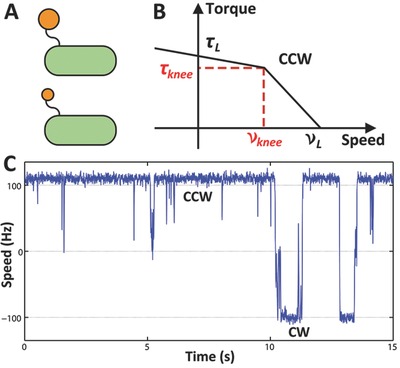
The output of the bacterial flagellar motor. A) A schematic plot of the bead rotation assay. In this assay, polystyrene beads of various sizes can be attached to the stub of a flagellum. B) The torque‐speed relationship of the motor rotating in CCW state. C) A typical 15‐ second speed trace of the motor.

Experimentally, the torque‐speed relationship of the BFM can be determined by attaching a polystyrene bead to the flagellum of a cell attaching to the surface of a glass coverslip (**Figure** **3**A). The rotation speed of the bead can be record by a fast camera mounted on a microscope while the viscosity of the external medium is rapidly changed by adding Ficoll,^4^ or while the drag coefficient of the bead is changed by varying the size of the bead.^5^

In Figure 3B, we see a typical torque‐speed relationship of the *E. coli* BFM. The torque (τ) of the motor remains approximately constant up to ca. 170 Hz (the “knee” velocity *v_knee_*), and then decreases sharply to zero at ca. 300 Hz. The sodium‐driven flagellar motor exhibits a similar relationship, but with a higher *v_knee_* and zero‐load speed. Previous studies and calculations have estimated that with high external load, the BFM converts almost all of the free energy released from the protons flow across into mechanical rotation of the load, indicating that the energy conversion efficiency of the BFM is very high.^7^, ^33^ Experiments that control the PMF show that the motor rotation speed depends linearly on the PMF in both low and high load regimes.^34^ The unusual shape of the torque‐speed relationship, the high energy conversion efficiency and PMF dependence are crucial to understanding the mechanism of the BFM, and thus receive extensive experimental and theoretical study.^3^, ^4^, ^35^, ^36^, ^37^

### 3.3 Stepping

The BFM has long been postulated to be a stepping motor, but only recently was experimental evidence found. Steps in the F1 ATPase, the only other rotary molecular motor, were first seen in 1998,^38^ and later, substeps were resolved.^39^ However, steps in BFM rotation were more difficult to resolve due to the high rotation speed and the small stepsize. In 2005, Sowa et al. constructed a chimera motor, with sodium driven stators in an *E. coli* BFM background.^40^ With this new motor, they managed to express only one stator under low sodium concentration. This resulted in a slow rotation rate, with long dwell time between steps, which aided step detection. An optical trapping system with high temporal and spatial resolution was used while a small indicator (a latex bead of diameter 0.2–0.5 microns) was attached to the flagellum. Finally, 26 steps per revolution were observed for the first time,^40^ consistent with the 26‐fold stoichiometry of the FliG protein on the C ring.

## 4 Switching of the Bacterial Flagellar Motor

An efficient method of propulsion alone is not enough to outcompete rivals and avoid toxins. A strategy for sensitive navigation is also required, and the BFM possesses this in bacterial chemotaxis. This process presents itself as the alternate “run” and “tumble” states of the cell. One important feature of *E. coli* chemotaxis is that the cell responds to temporal changes in attractant concentration while swimming, rather than spatial stimuli from a concentration gradient.^41^, ^42^ When *E. coli* runs up an attractant gradient, the run will be prolonged and the tumble be delayed.

The quick switching of the BFM between the “CCW” and “CW” states leads to the transition between “runs” and “tumbles” and forms the basis of bacterial chemotaxis. The switching of the BFM can be observed by the same bead rotation assay shown previously in measuring the torque‐speed relationship (Figure 3C).

This switching of the motor is finely controlled by a chemotactic sensory and signalling network. On the surface of the bacteria, there are arrays of chemoreceptors, methyl‐accepting chemotaxis proteins (MCPs), which detect environmental changes. Information from the environmental changes is translated into phosphorylation levels of a signaling molecule, CheY, through a series of biochemical reactions.

**Figure 4:**
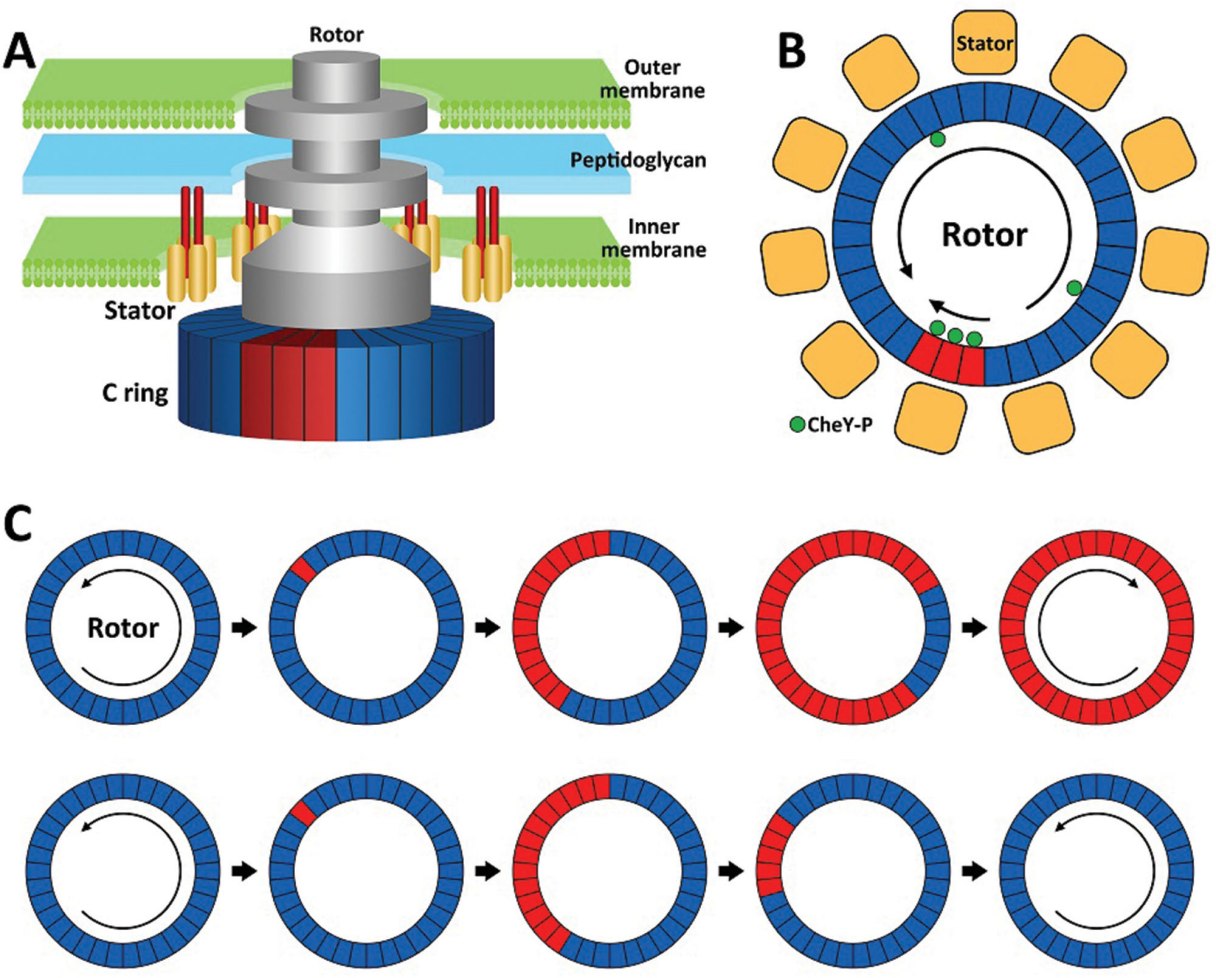
Conformational spread as a mechanism for ultrasensitivity in the flagellar motor switch. A) Some RSUs (red) are in the CW state while the others (blue) in the CCW state. B) Schematic top view of the motor, comprising a ring of 34 protomers and 11 stator units. C) The same‐state domain may grow to encompass the entire ring (top), known as conformational spread, or shrink and disappear as the motor restores its previous state (bottom).

The rotation bias of the motor is sensitively controlled by the cytoplasmic concentration of this small diffusible protein CheY‐P.^43^, ^44^ A lower concentration of CheY‐P in the cytoplasm results in more CCW rotation of the motor, while a higher concentration of CheY‐P causes more CW rotation. Previous works revealed that the interaction between CheY‐P molecules and the C‐ring is responsible for determining the BFM's direction of rotation.^45^, ^46^ Binding of CheY‐P to FliM is believed to trigger conformational changes in FliM, which is coupled to conformational changes in the FliG protein.^44^ Different structural orientation of the FliG protein generates two opposite directions of rotation (CCW or CW) when torque is delivered from the stator units (**Figure** **4**A).^47^

The switching of the BFM can be simplified by focusing on the interactions of the CheY‐P molecules with a ring‐shaped assembly of 34 rotor switching units (RSU), each of which is formed by 1 FliM, 1 FliN and ∼1 FliG proteins (Figure 4B). In this ring, each RSU is identical and can exist in either CCW or CW state, leading to CCW or CW rotation, respectively. One RSU can be bound or not bound to one CheY‐P molecule, and CheY‐P binding favors CW state. The motor spins full speed in CCW or CW while all RSUs are in a coherent state.^48^

The BFM of *E. coli* is remarkably sensitive: it can respond to attractant concentration as low as 1 μM.^33^, ^49^ To initiate a switch of the motor from CCW to CW rotation, some RSU units must bind a CheY‐P molecule. Previous experiments have revealed that the motor switching responses ultrasensitively to changes in CheY‐P concentration, meaning that only small changes in the number of CheY‐P bound on the ring can influence the switching state of the entire ring.^50^ To explain this ultrasensitivity, a “conformational spread” model has been proposed.^51^ In this model, a coupling energy between adjacent RSUs is introduced, where an energy penalty is applied if an RSU and its neighbors are in differing states. The existence of this coupling energy allows conformational spreading from one RSU to the rest, and successfully explains the experimental observation in which subtle changes in CheY‐P concentration can greatly change the switching bias of the motor. In the “conformational spread” model, a switching event of the motor usually starts with a switching event of a single RSU and this newly created domain may either grow to encompass the entire ring, or shrink and disappear (Figure 4C), as the ring returns to its previous coherent state.

Several key predictions of the conformational spread model have been validated by recent experiments,^49^, ^52^ which demonstrated that conformational spread is both necessary and sufficient to explain the switching mechanism of the BFM. As a generalization of the classical theories of allosteric regulation, conformational spread is applicable to any multimeric protein complex that responds to ligand binding. Thus the BFM utilizes the fundamental cooperativity of neighbor‐neighbor interactions to build a molecular gearbox that is capable of changing direction in milliseconds.

## 5 Structural Adaptivity of the Bacterial Flagellar Motor

What is remarkable about the sensitivity of the flagellar motor's switch complex is that it maintains a high sensitivity across a very large operating range. This sensitivity arises due to conformational spread as detailed above, but the operating range can be adjusted by directly adapting the structure of the rotor.^53^, ^54^ Yuan et al. showed that the number of FliM units can change in response to levels of CheY‐P, increasing the number of binding sites and thus sensitivity when CheY‐P concentrations are low. Furthermore, Lele et al. showed that FliM binding directly strengthened in response to the rotational direction of the motor, and that it was not CheY‐P binding that mattered, but only the direction of rotation. Indeed, not only the stators but both rotor proteins at the bottom of the rotor, FliM and FliN, have been shown to turnover during the motor operation.^55^, ^56^, ^57^, ^58^ This turnover is a property of bionanotechnology that lies in stark contrast to man‐made technology, that the structure of the motor can adaptively remodel, while operating, yet this seems to have evolved as a common theme throughout biology.^54^, ^59^, ^60^, ^61^ Thus far, turnover in FliG has not been observed,^62^ presumably due to its required role in torque‐generation and in the conformational change that dictates a switch event.^49^ This leads to a further perplexing symmetry mismatch between the top and bottom of the C‐ring: FliG should be ca. 34‐fold in *Salmonella enterica*,^63^ and while FliM is ca. 34‐fold in an exclusively clockwise rotating motor, it can be as many as 44‐fold in an exclusively counterclockwise rotating motor.^53^

Additionally, it has been shown recently that the motor can also adapts in response to increased load.^64^ Lele et al. showed here that the stators themselves dynamically respond to load, and this response was consistently observed even when chemotaxis and rotational switching was removed—it is the stators themselves that engage or disengage with changes in load. The similar observation has also been reported by Tipping et al.^58^ This is presumably because at low load only few stators are required to drive rotation and additional stators simply waste ions.^64^ However, the mechanism for force‐sensing of the stators remains unknown.

## 6 Perspective

The bacterial flagellar motor is the pinnacle of evolutionary bionanotechnology: a self‐assembling nanoscale electric rotary motor that performs at higher speed and with greater efficiency than any man‐made device. Study of such a machine, honed by billions of years of evolution, yields insight into the fundamental features of biological design. This insight, and the lessons from molecular architecture in general, can be harnessed to drive innovation in the creation of novel man‐made nanotechnology.

The BFM is already a good prototype for a self‐propelled nanobot. The steps in the chemotactic pathway of “detect”–“compare”–“decide”–“control motor” can be adopted to design signaling networks that govern motility in any synthetic device. Molecular motors have already been directly used in nanobiotechnology applications, for example, the use of cytoskeletal motor proteins for molecular transport of microtubules and actin filaments.^65^ Although these molecular‐motor powered devices are still in their infancy, they provide the possibility for designing nanomachines that can smartly deliver drugs to a target guided only by extracellular signals.

However, the BFM offers not only impressive mechanical performance and desirable design traits, it also contains a simple switching mechanism that allows the motor to change direction quickly. This mechanism, based upon conformational change, is an example of complex behavior arising from simple neighbor‐neighbor interactions, and highlights a fundamental difference in the way biological machines function. Man‐made nanotechnology typically is designed brick by brick with an architectural plan from above. Evolution, in contrast, favours the emergence of complex behavior from simple, tunable interactions.^66^ By studying how the BFM rotates and switches, how it assembles, and how it arose, we enable the next era of nanotechnology in which we apply these natural design principles in the creation of new synthetic biological machinery.

## References

[1] H. C. Berg , E. coli in Motion, Springer‐Verlag, New York, 2004.

[2] G. Lowe , M. Meister , H. C. Berg , Nature 1987, 325, 637.

[3] W. S. Ryu , R. M. Berry , H. C. Berg , Nature 2000, 403, 444. 10667798 10.1038/35000233

[4] X. Chen , H. C. Berg , Biophys. J. 2000, 78, 1036. 10653817 10.1016/S0006-3495(00)76662-8 PMC1300707

[5] Y. Sowa , H. Hotta , M. Homma , A. Ishijima , J. Mol. Biol. 2003, 327, 1043. 12662929 10.1016/s0022-2836(03)00176-1

[6] J. Xing , F. Bai , R. Berry , G. Oster , Proc. Natl. Acad. Sci. USA 2006, 103, 1260. 16432218 10.1073/pnas.0507959103 PMC1360542

[7] H. C. Berg , Annu. Rev. Biochem. 2003, 72, 19. 12500982 10.1146/annurev.biochem.72.121801.161737

[8] J. Adler , W. W. Tso , Science 1974, 184, 1292. 4598187 10.1126/science.184.4143.1292

[9] D. A. Rasko , V. Sperandio , Nat. Rev. Drug Discov. 2010, 9, 117. 20081869 10.1038/nrd3013

[10] D. J. Clark , O. Maaløe , J. Mol. Biol. 1967, 23, 99.

[11] M. Riley , T. Abe , M. B. Arnaud , M. K. Berlyn , F. R. Blattner , R. R. Chaudhuri , J. D. Glasner , T. Horiuchi , I. M. Keseler , T. Kosuge , H. Mori , N. T. Perna , G. Plunkett 3rd , K. E. Rudd , M. H. Serres , G. H. Thomas , N. R. Thomson , D. Wishart , B. L. Wanner , Nucleic Acids Res. 2006, 34, 1. 16397293 10.1093/nar/gkj405 PMC1325200

[12] F. R. Blattner , G. Plunkett 3rd , C. A. Bloch , N. T. Perna , V. Burland , M. Riley , J. Collado‐Vides , J. D. Glasner , C. K. Rode , G. F. Mayhew , J. Gregor , N. W. Davis , H. A. Kirkpatrick , M. A. Goeden , D. J. Rose , B. Mau , Y. Shao , Science 1997, 277, 1453. 9278503 10.1126/science.277.5331.1453

[13] F. A. Samatey , K. Imada , S. Nagashima , F. Vonderviszt , T. Kumasaka , M. Yamamoto , K. Namba , Nature 2001, 410, 331. 11268201 10.1038/35066504

[14] K. Yonekura , S. Maki‐Yonekura , K. Namba , Nature 2003, 424, 643. 12904785 10.1038/nature01830

[15] L. Turner , W. S. Ryu , H. C. Berg , J. Bacteriol. 2000, 182, 2793. 10781548 10.1128/jb.182.10.2793-2801.2000 PMC101988

[16] M. L. DePamphilis , J. Adler , J. Bacteriol. 1971, 105, 384. 4993325 10.1128/jb.105.1.384-395.1971 PMC248366

[17] F. A. Samatey , H. Matsunami , K. Imada , S. Nagashima , T. Shaikh , R. Thomas , D. R. Chen , J. Z. Derosier , D. J. Kitao , A. Namba , K. , Nature 2004, 431, 1062. 15510139 10.1038/nature02997

[18] Y. Sowa , R. M. Berry , Q. Rev. Biophys. 2008, 41, 103. 18812014 10.1017/S0033583508004691

[19] D. F. Blair , FEBS Lett. 2003, 545, 86. 12788496 10.1016/s0014-5793(03)00397-1

[20] T. F. Braun , L. Q. Al‐Mawsawi , S. Kojima , D. F. Blair , Biochemistry 2004, 43, 35. 14705929 10.1021/bi035406d

[21] J. Zhou , S. A. Lloyd , D. F. Blair , Proc. Natl. Acad. Sci. USA 1998, 95, 6436. 9600984 10.1073/pnas.95.11.6436 PMC27776

[22] J. Zhou , D. F. Blair , J. Mol. Biol. 1997, 273, 428. 9344750 10.1006/jmbi.1997.1316

[23] S. A. Lloyd , D. F. Blair , J. Mol. Biol. 1997, 266, 733. 9102466 10.1006/jmbi.1996.0836

[24] J. Zhou , L. L. Sharp , H. L. Tang , S. A. Lloyd , S. Billings , T. F. Braun , D. F. Blair , J. Bacteriol. 1998, 180, 2729. 9573160 10.1128/jb.180.10.2729-2735.1998 PMC107227

[25] T. F. Braun , S. Poulson , J. B. Gully , J. C. Empey , S. Van Way , A. Putnam , D. F. Blair , J. Bacteriol. 1999, 181, 3542. 10348868 10.1128/jb.181.11.3542-3551.1999 PMC93823

[26] R. M. Macnab , Annu. Rev. Microbiol. 2003, 57, 77. 12730325 10.1146/annurev.micro.57.030502.090832

[27] T. Minamino , K. Namba , J. Mol. Microbiol. Biotechnol. 2004, 7, 5. 15170399 10.1159/000077865

[28] K. Yonekura , S. Maki , D. G. Morgan , D. J. DeRosier , F. Vonderviszt , K. Imada , K. Namba , Science 2000, 290, 2148. 11118149 10.1126/science.290.5499.2148

[29] L. D. Evans , S. Poulter , E. M. Terentjev , C. Hughes , G. M. Fraser , Nature 2013, 504, 287. 24213633 10.1038/nature12682 PMC3864836

[30] T. Minamino , K. Imada , K. Namba , Mol. Biosyst. 2008, 4, 1105. 18931786 10.1039/b808065h

[31] H. C. Berg , Random Walks in Biology, Princeton University Press, Princeton, 1993.

[32] E. M. Purcell , Am. J. Phys. 1977, 45, 3.

[33] R. M. Berry , in Encyclopedia of Life Sciences, Nature Publishing Group London 2001.

[34] C. V. Gabel , H. C. Berg , Proc Natl Acad Sci USA 2003, 100, 8748. 12857945 10.1073/pnas.1533395100 PMC166384

[35] H. C. Berg , L. Turner , Biophys. J. 1993, 65, 2201. 8298044 10.1016/S0006-3495(93)81278-5 PMC1225952

[36] R. M. Berry , H. C. Berg , Biophys. J. 1999, 76, 580. 9876171 10.1016/S0006-3495(99)77226-7 PMC1302548

[37] J. Yuan , H. C. Berg , Biophys. J. 2010, 98, 2121. 20483319 10.1016/j.bpj.2010.01.061 PMC2872210

[38] R. Yasuda , H. Noji , K. Kinosita Jr. , M. Yoshida , Cell 1998, 93, 1117. 9657145 10.1016/s0092-8674(00)81456-7

[39] R. Yasuda , H. Noji , M. Yoshida , K. Kinosita Jr. , H. Itoh , Nature 2001, 410, 898. 11309608 10.1038/35073513

[40] Y. Sowa , A. D. Rowe , M. C. Leake , T. Yakushi , M. Homma , A. Ishijima , R. M. Berry , Nature 2005, 437, 916. 16208378 10.1038/nature04003

[41] J. E. Segall , S. M. Block , H. C. Berg , Proc. Natl. Acad. Sci. USA 1986, 83, 8987. 3024160 10.1073/pnas.83.23.8987 PMC387059

[42] D. A. Brown , H. C. Berg , Proc. Natl. Acad. Sci. USA 1974, 71, 1388. 4598304 10.1073/pnas.71.4.1388 PMC388234

[43] M. Welch , K. Oosawa , S. Aizawa , M. Eisenbach , Proc. Natl. Acad. Sci. USA 1993, 90, 8787. 8415608 10.1073/pnas.90.19.8787 PMC47445

[44] A. S. Toker , R. M. Macnab , J. Mol. Biol. 1997, 273, 623. 9356251 10.1006/jmbi.1997.1335

[45] P. Cluzel , M. Surette , S. Leibler , Science 2000, 287, 1652. 10698740 10.1126/science.287.5458.1652

[46] D. Bray , Proc. Natl. Acad. Sci. USA 2002, 99, 7.11782543

[47] L. K. Lee , M. A. Ginsburg , C. Crovace , M. Donohoe , D. Stock , Nature 2010, 466, 996. 20676082 10.1038/nature09300 PMC3159035

[48] Q. Ma , D. V. Nicolau Jr. , P. K. Maini , R. M. Berry , F. Bai , PLoS Comput. Biol. 2012, 8, e1002523. 22654654 10.1371/journal.pcbi.1002523 PMC3359969

[49] F. Bai , R. W. Branch , D. V. Nicolau Jr. , T. Pilizota , B. C. Steel , P. K. Maini , R. M. Berry , Science 2010, 327, 685. 20133571 10.1126/science.1182105

[50] V. Sourjik , H. C. Berg , Mol Microbiol 2000, 37, 740. 10972797 10.1046/j.1365-2958.2000.02044.x

[51] T. A. Duke , N. Le Novere , D. Bray , J. Mol. Biol. 2001, 308, 541. 11327786 10.1006/jmbi.2001.4610

[52] F. Bai , T. Minamino , Z. Wu , K. Namba , J. Xing , Phys. Rev. Lett. 2012, 108, 178105. 22680910 10.1103/PhysRevLett.108.178105 PMC3558881

[53] P. P. Lele , R. W. Branch , V. S. Nathan , H. C. Berg , Proc. Natl. Acad. Sci. USA 2012, 109, 20018. 23169659 10.1073/pnas.1212327109 PMC3523824

[54] J. H. Yuan , R. W. Branch , B. G. Hosu , H. C. Berg , Nature 2012, 484, 233. 22498629 10.1038/nature10964 PMC3335734

[55] N. J. Delalez , R. M. Berry , J. P. Armitage , MBio 2014, 5, e01216. 24987089 10.1128/mBio.01216-14 PMC4161238

[56] N. J. Delalez , G. H. Wadhams , G. Rosser , Q. Xue , M. T. Brown , I. M. Dobbie , R. M. Berry , M. C. Leake , J. P. Armitage , Proc. Natl. Acad. Sci. USA 2010, 107, 11347. 20498085 10.1073/pnas.1000284107 PMC2895113

[57] N. Delalez , J. P. Armitage , Mol. Microbiol. 2009, 71, 807. 19170878 10.1111/j.1365-2958.2008.06573.x

[58] M. J. Tipping , N. J. Delalez , R. Lim , R. M. Berry , J. P. Armitage , MBio 2013, 4, e00551. 23963182 10.1128/mBio.00551-13 PMC3747592

[59] A. Loquet , N. G. Sgourakis , R. Gupta , K. Giller , D. Riedel , C. Goosmann , C. Griesinger , M. Kolbe , D. Baker , S. Becker , A. Lange , Nature 2012, 486, 276. 22699623 10.1038/nature11079 PMC3598588

[60] A. S. Olia , P. E. Prevelige Jr. , J. E. Johnson , G. Cingolani , Nat. Struct. Mol. Biol. 2011, 18, 597. 21499245 10.1038/nsmb.2023 PMC3087855

[61] D. Stock , A. G. Leslie , J. E. Walker , Science 1999, 286, 1700. 10576729 10.1126/science.286.5445.1700

[62] H. Fukuoka , Y. Inoue , S. Terasawa , H. Takahashi , A. Ishijima , Biochem. Biophys. Res. Commun. 2010, 394, 130. 20184859 10.1016/j.bbrc.2010.02.129

[63] D. DeRosier , Curr. Biol. 2006, 16, R928. 17084692 10.1016/j.cub.2006.09.053

[64] P. P. Lele , B. G. Hosu , H. C. Berg , Proc. Natl. Acad. Sci. USA 2013, 110, 11839. 23818629 10.1073/pnas.1305885110 PMC3718179

[65] M. G. van den Heuvel , C. Dekker , Science 2007, 317, 333. 17641191 10.1126/science.1139570

[66] S. A. Kauffman , The Origins of Order. Self‐Organization and Selection in Evolution, Oxford University Press, New York 1993.

